# Lunge feeding in early marine reptiles and fast evolution of marine tetrapod feeding guilds

**DOI:** 10.1038/srep08900

**Published:** 2015-03-10

**Authors:** Ryosuke Motani, Xiao-hong Chen, Da-yong Jiang, Long Cheng, Andrea Tintori, Olivier Rieppel

**Affiliations:** 1 Department of Earth and Planetary Sciences, University of California, Davis, One Shields Avenue, Davis, California 95616, U.S.A.; 2 Wuhan Centre of China Geological Survey, Wuhan, Hubei 430023, P. R. China; 3 Laboratory of Orogenic Belt and Crustal Evolution, Ministry of Education; Department of Geology and Geological Museum, Peking University, Yiheyuan Street. 5, Beijing 100871, P.R. China; 4 Dipartimento di Scienze della Terra, Università degli Studi di Milano, Via Mangiagalli 34-20133 Milano, Italy; 5 Center of Integrative Research, The Field Museum, Chicago. IL 60605-2496, U.S.A

## Abstract

Traditional wisdom holds that biotic recovery from the end-Permian extinction was slow and gradual, and was not complete until the Middle Triassic. Here, we report that the evolution of marine predator feeding guilds, and their trophic structure, proceeded faster. Marine reptile lineages with unique feeding adaptations emerged during the Early Triassic (about 248 million years ago), including the enigmatic *Hupehsuchus* that possessed an unusually slender mandible. A new specimen of this genus reveals a well-preserved palate and mandible, which suggest that it was a rare lunge feeder as also occurs in rorqual whales and pelicans. The diversity of feeding strategies among Triassic marine tetrapods reached their peak in the Early Triassic, soon after their first appearance in the fossil record. The diet of these early marine tetrapods most likely included soft-bodied animals that are not preserved as fossils. Early marine tetrapods most likely introduced a new trophic mechanism to redistribute nutrients to the top 10 m of the sea, where the primary productivity is highest. Therefore, a simple recovery to a Permian-like trophic structure does not explain the biotic changes seen after the Early Triassic.

Biotic changes after the end-Permian extinction have been studied extensively in recent years, especially in terms of biotic carbon fluctuation, taxonomic diversity, and trophic structure recovery^1^,^2^. A prevailing view is that the post-extinction recovery was slow and gradual^2^, despite some changes in the taxonomic composition of the predator guild^3^. However, the change in marine trophic structure during the earlier half of the Triassic was probably more than a simple recovery to the previous Late Permian structure. Marine tetrapods, which later gave rise to important top predators^4^, emerged in the Early Triassic (see supplementary note for a discussion of mesosaurs). Marine tetrapods move vertically between their feeding and resting habitats because they need to breathe and bask at the sea surface after feeding in deeper waters. Their defecation near the surface pumps nutrients from deeper feeding habitats to the top 10 m of the water column, which usually has the highest primary productivity due to higher light levels^5^. Such nutrient redistribution is exemplified by nitrogen cycling by marine mammals along the North Atlantic coast^6^, as well as iron circulation by marine tetrapods in the Southern Ocean^7^. The emergence of this novel nutrient-cycling mechanism in the Triassic may mark the onset of a shift in the trophic structure of coastal seawaters, although its net effect may have been small initially depending on the total metabolic rates of marine tetrapods in a given region. It is therefore important to closely examine the evolution of marine tetrapods in the earlier half of the Triassic to illuminate any changes in marine trophic structure during the recovery phase from the end-Permian mass extinction.

Three major lineages of marine tetrapods are generally recognized in the Early and Middle Triassic, namely Ichthyopterygia^8^, Sauropterygia^9^, and Thalattosauriformes^10^. The number of marine invasions by reptiles at the time is controversial, and depends on the scheme of phylogenetic relationships adopted^11^. However, phylogenetic diversity is not directly relevant to the question of trophic structures. Instead, it is the variation in feeding styles among these marine tetrapods that is more indicative of the trophic structures of the time. Apart from these three major lineages, several minor lineages of marine reptiles were also present, each of which possessed unique suites of feeding adaptations that persisted for only short time spans. Some of these often-ignored groups are poorly understood, concealing the true diversity of feeding styles among early marine tetrapods. It is essential to examine the feeding functions of these reptiles in order to provide the raw data for a comprehensive analysis of feeding style diversity. One of the most problematic of these enigmatic lineages has been the Hupehsuchia^12^, the sister taxon of Ichthyosauriformes^13^.

Hupehsuchians lived during the latest Early Triassic (about 247.6 million years ago), and so far exclusively in what is now the north-central part of Hubei Province, China. Despite their limited temporal and spatial distribution, the generic diversity of the group was high, with five genera currently recognized^12^,^14^,^15^,^16^. They ranged from ~0.4 to ~2.0 m in adult body length, and had a heavily built trunk covered by dorsal dermal ossicles and overlapping gastralia. One of the most peculiar features of the group is the skull, which has a flattened, edentulous rostrum that is superficially reminiscent of a duck's bill. Its mandibular rami are unusually slender for a vertebrate. The peculiar rostral morphology of *Hupehsuchus* has led to uncertainty about its feeding mode. Based on the general configuration of the rostrum, it was suggested that *Hupehsuchus* was similar to some baleen whales and therefore may have had baleens-like structures although no impressions of such structures have been found on the palate^17^. However, the resemblance to baleen whales was questioned by the same authors who found the neck to be excessively long, and the skull too small and narrow for whale-like lunge feeding^17^. The latter view was supported by later researchers^18^, leaving the feeding style of *Hupehsuchus* ambiguous.

Recent fieldwork by the Wuhan Centre of China Geological Survey (WGSC) has yielded many marine tetrapod specimens from the Lower Triassic, revealing a surprising diversity of these early marine invaders. New taxa have been described and hupehsuchian phylogeny reconstruction has been substantially improved^11^,^15^. The fieldwork also recovered the well-preserved specimen of *Hupehsuchus* reported herein, revealing hitherto unknown morphology, particularly with regard to the palate and mandible (Fig. 1). The specimen (Wuhan Centre of China Geological Survey, WGSC V26000) closely resembles *Hupehsuchus nanchangensis* except in a few minor differences in vertebral count, phalangeal formula, and longitudinal spacing of limb elements. While these differences may suggest that the specimen may belong to a new species, we remain conservative and refer to it as *Hupehsuchus* sp. until more comparative data are available.

## Feeding function of *Hupehsuchus*

The new specimen shows that the skull and mandible of *Hupehsuchus* is characterized by a mixture of features resembling feeding adaptations in pelicans and rorqual whales, suggesting that the genus shared the feeding style of these two animals. Pelicans, rorquals, and pelican eels share a common feeding style of capturing prey in a gular pouch together with a large amount of water as they move forward, while their flexible jaw symphyses permit expansion of the lower jaw^19^,^20^,^21^. However, there are differences among these groups in terms of the mechanisms of gular space widening and in how they eliminate excess water from the buccal cavity. Notably, the gular space in pelicans is widened by mandibular bowing and rotation, which is facilitated by the flexibility of the mandibular rami and the jaw symphysis^22^. By contrast, rorquals use rotation of the solid mandibular rami alone^19^. The pelican eel differs from the other two in expanding both the mandible and the upper jaw^21^. For convenience, we will hereafter refer to the feeding styles of pelicans and the pelican eel as lunge feeding, as is also characteristic of rorquals (see Methods). To examine the resemblance between *Hupehsuchus* and extant lunge feeders, we will focus on the following three questions. First, does the mandibular shape of *Hupehsuchus* imply pelican-like bowing that would enable lunge feeding? Second, is the head of *Hupehsuchus* too small in comparison with those of lunge-feeding whales to permit lunge feeding, as has been suggested before? Third, is there any structure that suggests the presence of a strainer in the mouth cavity of *Hupehsuchus*?

### Mandible shape

Many major vertebrate groups include species with slender jaws, such as the pelican eel (*Eurypharynx pelecanoides*)^21^,^23^, longnose butterflyfish (*Forcipiger longirostris*)^24^,^25^, longnose gar (*Lepisosteus osseous*)^26^, needlefishes (Belonidae)^23^,^27^, and halfbeaks (Hemirhamphidae)^28^ among bony fishes, as well as hummingbirds (Trochilidae)^29^, curlews (*Numenius* spp.)^30^, pelicans (*Pelecanus* spp.)^22^, and ibis and spoonbills (Threskiornithidae)^31^ among birds (Fig. 2). The mandible of *Hupehsuchus* remains one of the most slender among vertebrates even in comparison with these species. Notably, ducks have robust mandibles that are unlike those of *Hupehsuchus* despite the superficial resemblance of snout outline between both groups in palatal view (Fig. 2). The mandibular symphysis of *Hupehsuchus* is loose as in extant lunge feeders, and the two rami are widely separated from each other as in pelicans^22^. In other slender-jawed species, the two rami are usually close together; even if they are not, there is a robust symphysis (as in spoonbills).

We calculated the relative mandibular depth distribution along the jaw for 30 selected vertebrates, most of which are known for their slender jaws (see Methods). The result shows that the flexural rigidity inferred from mandibular depths is distributed along the jaw similarly in *Hupehsuchus* (Fig. 3A, black line) and the brown pelican (blue line). Mandibular depth and flexural rigidity are closely tied because if one deepens the jaw by a factor of two while not changing other dimensions, the dorsoventral flexural rigidity is expected to increase by a factor of eight according to beam theory. A geometric morphometric analysis of the mandibular outline (see Methods) suggests that the mandible of *Hupehsuchus* is similar to those of extant lunge feeders, namely the brown pelican and the pelican eel (Fig. 3C, D). These two extant lunge feeders and *Hupehsuchus* have the most slender jaws of the vertebrates that we examined (Fig. 2).

Slender mandibles are generally known to flex for various functions^22^,^32^,^33^,^34^, but when combined with a flexible jaw symphysis, these mandibles seem to be used for lunge feeding as in pelicans and the pelican eel. The broad separation between the two mandibular rami in *Hupehsuchus* suggests a soft tissue membrane bridging the two. Such a membrane between the widely-separated mandibular rami can form a gular pouch when combined with slender jaws, as in the pelican eel^21^, pelicans^22^ and spoonbills^32^. However, spoonbills are not the best analog for *Hupehsuchus* because their mandibular rami are wide (although slender in lateral view) and form a solid spoon-shaped symphysis for grasping. When combining the information above, it appears that the mandible of *Hupehsuchus* was designed to bow as in pelicans, with a gular pouch between its two rami.

### Mandible size

Previous studies pointed out that baleen whales had larger skulls relative to body size than *Hupehsuchus*, although without explicit quantification^17^,^18^. According to a more recent dataset^35^, the mandibular ratio (mandible length divided by body length) ranges between 18.9 and 26.7% in Balaenopteridae, and between 17.1 and 31.9% in Mysticeti, while our new and complete specimen of *Hupehsuchus* suggests a ratio of 14.7%. Given the large difference in body size between balaenopterid whales and *Hupehsuchus*, the latter may have had an equally large mandible for its body size. We therefore examined the allometry of mandibular length in rorqual whales to find out the expected mandibular size for a hypothetical miniature rorqual with the body length of *Hupehsuchus*.

A phylogenetic generalized least square (PGLS) regression suggested slightly positive allometry of mandibular length against body length in rorqual whales (slope = 1.07, intercept = −0.728) (Fig. 3E). A standardized major axis (SMA) regression based on phylogenetically independent contrast (PIC) also suggested similar regression coefficients (1.08, −0.737; PIC does not provide the intercept, which was instead calculated from the slope and phylogenetic means). The PGLS and SMA-PIC regression lines are visually indistinguishable at the scale of Figure 3E, and so is the non-phylogenetic regression line based on ordinary least square (OLS). The slope is slightly different from the value of 1.11 that was originally reported^35^ largely because of the difference in the phylogenetic hypothesis used in the calculation—the original phylogeny had uniform branch lengths throughout, while our estimate is based on published divergence time estimates (see Methods). An extrapolation from the allometric regression (Fig. 3E, dashed line) suggests that the mandible of *Hupehsuchus* is slightly smaller than that expected for a hypothetical rorqual whale with the same body length as *Hupehsuchus*, but the difference is minor. It is difficult to assess if *Hupehsuchus* falls within the range predicted from rorqual whales in a phylogenetic framework because PGLS does not provide confidence intervals, as with GLS in general. However, given that there is little difference between PGLS, SMA-PIC, and OLS lines, phylogenetic bias contained in this regression is probably minimal, justifying the use of non-phylogenetic prediction intervals from OLS. When superimposing the OLS prediction interval on Fig. 3E, it suggests that *Hupehsuchus* lies within the range predicted from balaenopterids. Note that the comparison assumes mammalian metabolic and food-consumption rates but *Hupehsuchus*, with its reptilian rates, likely could do with smaller jaws than predicted in Fig. 3E (see Supplementary Information).

### Palatal structure

**Figure 1:**
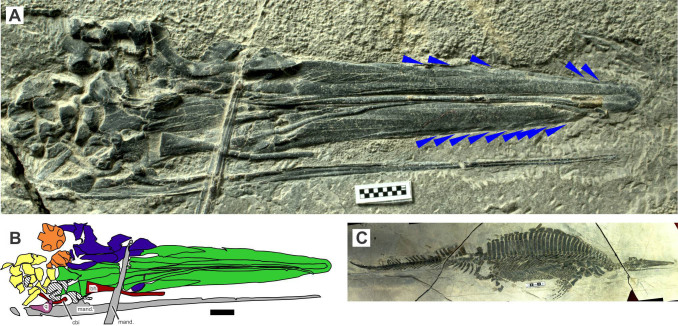
New specimen of *Hupehsuchus* sp. (WGSC V26000). (A), palatal view of the skull; (B), colour map of (A); (C), whole body. Scales are in centimeters. Colours in (B) are: blue, skull roof and sides; brown, hyobranchial elements; green, palate; orange, occiput; yellow, postcranium. Hatched bones are unidentified. Abbreviations: bh, basihyal; cbi, ceratobranchial I; mand, mandibular ramus; q, quadrate. Blue triangles point to the intersection between the jaw margin and the shallow groove-like depressions, probably indicating the presence of a soft-tissue structure. Photographs were taken by R.M.

The new specimen, which is the first to preserve the details of the dermal palate, revealed that the palate was not completely flat but convex (Fig. 1)—the jaw margin curls up dorsally, while the area along the sagittal plane bulges ventrally. This condition is reminiscent of the upper jaws of pelicans and spoonbills^32^. The jaw margin is not smooth but instead has a series of oblique parallel depressions, oriented from rostromedially to caudolaterally. The angle between the sagittal plane and these shallow groove-like depressions is approximately 15–25°. Each depression is up to ~1 mm wide, and around three depressions intersect each 1 cm section of the jaw margin. Their medial extent seems to be limited within a 2 mm-wide belt that is parallel to the jaw margin. Only the premaxilla seems to bear these depressions. The grooves are reminiscent of the fluting seen in the palatal margins of some *Balaenoptera* specimens, although these rorqual structures extend much more medially and the direction is mirrored caudorostrally. The palatal grooves of *Hupehsuchus* are evident only under specific light conditions. However, their presence is detectable by touching the surface. The depressions are most likely impressions left by the soft tissues that were appressed to the upper jaw margin.

Given the groove-like palatal depressions along the jaw margin, it is likely that some soft tissue structures were present along the palatal margin of *Hupehsuchus*. Whether it was a structure resembling the pecten of ducks or the baleen of rorquals remains unknown. Yet, such a structure is functionally positioned to strain the water as it was expelled from the mouth cavity. Supporting this suggestion is the presence of a robust entoglossal process of the hyoid body, which is present in extant reptiles with large tongues^36^. Such a tongue would have been useful in actively expelling the water from the mouth cavity through the strainer. Soft-tissue fossils would be necessary to test such an inference.

**Figure 2:**
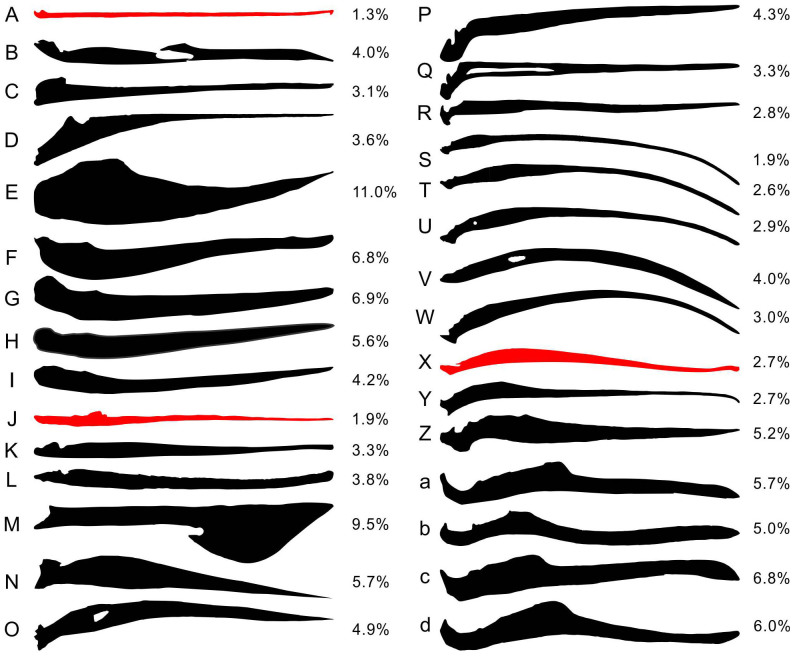
Selected slender mandibles of vertebrates drawn to same length, with some robust jaws for comparison. (A), pelican eel (*Eurypharynx pelecanoides*); (B), longnose butterflyfish (*Forcipiger longirostris*); (C), longnose gar (*Lepisosteus* *osseous*); (D), New Zealand piper (*Hyporhamphus ihi*); (E), *Cartorhynchus* *lenticarpus*; (F), *Cymbospondylus piscosus*; (G), *Temnodontosaurus platyodon*; (H), *Ophthalmosaurus icenicus*; (I), *Chaohusaurus chaoxianensis*; (J), *Hupehsuchus* sp.; (K), *Stomatosuchus inermis*; (L), *Laganosuchus thaumastos*; (M), *Ikrandraco avatar*; (N), sandhill crane (*Grus canadensis*); (O), limpkin (*Aramus guarauna*); (P), American woodcock (*Philohela minor*); (Q), Wilson's snipe (*Gallinago delicate*); (R), black-necked stilt (*Himantopus mexicanus*); (S), long-billed curlew (*Numenius americanus*); (T), whimbrel (*Numenius phaeopus*); (U), Eskimo curlew (*Numenius borealis*); (V), purple-throated carib (*Eulampis jugularis*); (W), scarlet ibis (*Eudocimus ruber*); (X), brown pelican (*Pelecanus occidentalis*); (Y), roseate spoonbill (*Platalea ajaja*); (Z), Eurasian oystercatcher (*Haematops ostralegus*); (a), black-billed whistling duck (*Dendrocygna autumnalis*); (b), northern shoveler (*Spatula clypeata*); (c), white-backed duck (*Thalassornis leuconotus*); (d), ruddy duck (*Oxyura jamaicensis*). (A–D), bony fish; (E–M) fossil reptiles; (N–d), birds. A close similarity in slenderness exists among pelicans, the pelican eel, and *Hupehsuchus* (red color); they also have flexible jaw symphyses. Associated percentage values are the mean depths of the mandibles relative to respective lengths (see Methods). See Supplementary Note for figure sources.

**Figure 3:**
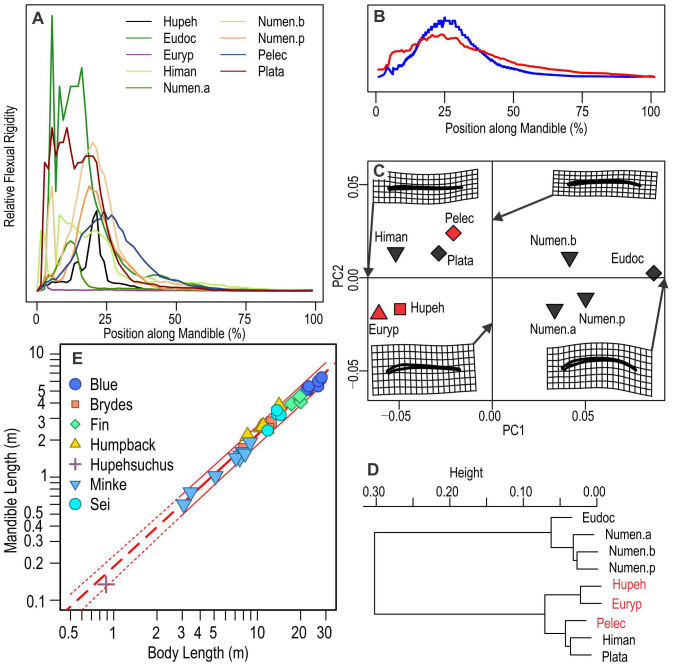
Comparison of jaw shape and flexural rigidity. (A), distribution of relative flexural rigidity along the mandible, based on the biquadratic of relative depths; (B), comparison of estimated relative flexural rigidity (blue) with a published curve (red) in the brown pelican; (C), first two axes of PCA from a geometric morphometric analysis; (D), hierarchical cluster analysis of data used in (C); (E), phylogenetic regression of mandible versus body length in rorqual whales, with extrapolation for *Hupehsuchus*. Symbols in (C): diamond, Pelecaniformes; reverse triangle, Scolopacidae; square, *Hupehsuchus*; and triangle, Osteichthyes. (A), (C), and (D) demonstrate a close similarity between extant lunge feeders and *Hupehsuchus* in jaw shape and the flexibility it implies. Red text highlights taxa that are judged to be lunge feeders. Thick red regression line in (E) is based on PGLS through species means, although points represent individual specimens. Thin curves associated with the PGLS line are based on OLS regression. See text and Methods.

The upper jaw of *Hupehsuchus* may resemble some duck bills in outline but the two differ greatly in dorsoventral topology; the palate bulges ventrally along the sagittal plane in *Hupehsuchus* as in pelicans and rorquals but unlike in the condition present in ducks. Combined with the drastic difference in mandibular morphology (Figs. 2, 3), duck-like feeding is implausible for *Hupehsuchus*.

### Other structures

Long necks and slender skulls have been suggested as counter-evidence against suspension-feeding (including lunge feeding) in *Hupehsuchus*^17^,^18^. However, it is obvious that neither is important. Pelicans have necks much longer than those of *Hupehsuchus* yet are capable of lunge feeding. Pelican skulls are narrow, but again this narrowness does not interfere with their lunge feeding. Hupehsuchians had a well-developed retroarticular process of the mandible, unlike Ichthyosauriformes. A long retroarticular process improves the mechanical advantage of jaw depressor muscles, indicating an emphasis on efficient jaw opening during feeding, which is also consistent with lunge feeding.

### Conclusion

To summarize, *Hupehsuchus* was most likely a lunge feeder that could widen the mandible to expand a gular pouch through mandibular bowing, based on the inferred flexibility of the mandible. However, unlike pelicans, it probably expelled water from the mouth cavity using a large tongue and through a soft tissue strainer along the jaw margin. Whether expulsion occurred under- or above water is unknown. Despite previous suggestions, its head was not too small for a lunge feeder of its size, especially given an inferred reptilian metabolic rate. Similarly, its neck was not too long or its skull too narrow, to be a lunge feeder. Its limbs and tail were well developed to generate thrust for acceleration and maneuvering. It was probably a demersal feeder given its heavy skeleton, but collisions of the mandible with substrates during lunging can be avoided by adjusting the trajectory of lunging. *Hupehsuchus* most likely represents the first lunge feeder in the history of life, appearing about 247.6 million years ago in the Spathian (late Early Triassic), more than 200 million years before rorqual whales or pelicans (see Methods).

## Trophic implications of marine tetrapod feeding guilds in the Triassic

### Feeding style diversity through the Triassic

As mentioned earlier, Hupehsuchia was not the only marine reptile group that appeared after the end-Permian mass extinction to possess an innovative feeding strategy. For example, the recently described *Cartorhynchus*, also from the Spathian, was the first and only suction-feeder among Ichthyosauriformes^13^,^37^, and probably among all Triassic marine reptiles—no other marine reptile possesses the suite of hyoid and rostral features listed by previous studies as mechanically important in suction feeding^37^, especially the narrowing of the rostrum that allows suction pressure concentration as in syringes. The enigmatic *Omphalosaurus*, again appearing in the Spathian, also had a strange jaw design with rounded teeth located far rostrally from the jaw joint along an elongate mandibular symphysis, with the rest of the jaw being edentulous^38^,^39^. Despite the shape of the teeth, their position and the low mechanical advantage present at the front of the jaws demonstrates that durophagy was unlikely. Instead, the extensive jaw symphysis seems to form a grasping structure similar to the spoon of spoonbills^39^,^40^. Accordingly, *Omphalosaurus* most likely grasped food with its rounded teeth. *Atopodentatus* from the Anisian (early Middle Triassic) was a filter-feeder^41^. Notably, some of these unique feeding styles are analogous to those of some modern avian adaptations: birds have not usually been considered as modern analogs for Triassic marine reptiles.

**Figure 4:**
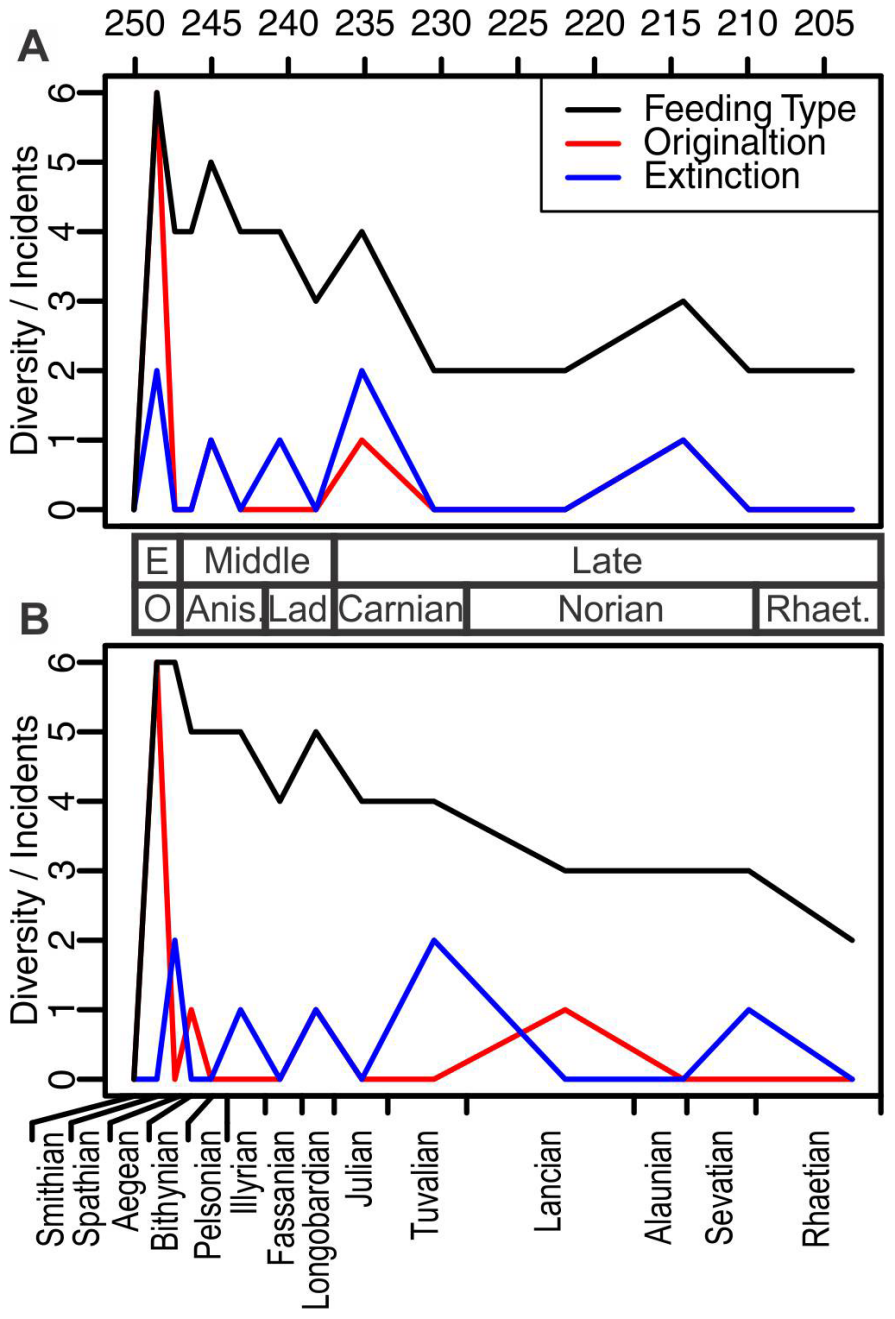
Feeding type diversity of marine tetrapods through the Triassic. (A), raw data from Supplementary Table S1; (B), modified data to account for the incompleteness of fossil record. See text. Both graphs show the burst of feeding types in the Early Triassic, followed by a decline. Numbers on the top represent the absolute age (million years ago). Names between (A) and (B) are epochs and ages of the Triassic, and those at the bottom are substages.

We divided the feeding strategies of Triassic marine reptiles into 24 categories based on three features concerning prey selection, capture and processing that can be assessed using fossils, namely the feeding habitat (pelagic or demersal), prey capture (ram with biting, lunge, or suction), and tooth shape (pointed, rounded, filter, or edentulous); see Methods for details of categorization). These three factors are expected to indicate differences in prey preference among these marine reptiles^42^,^43^. The resulting feeding categories may be further subdivided by adding more criteria. However, such a complicated model may obscure the basic pattern by emphasizing the details. We therefore decided to use the three basic factors to capture any large-scale patterns in the evolution of feeding guilds among Triassic marine tetrapods. About half of the 24 are unlikely to be occupied, e.g., teeth are not expected in suction or lunge feeders. In the present case, 15 of the 24 were unoccupied and subsequently removed from the list. Occupancy of the remaining nine categories through time is summarized in Supplementary Table S1. Notably, the results show that the highest variation in marine tetrapod feeding strategy was reached in the Spathian (Fig. 4), despite the fact that marine reptiles most likely did not emerge until the mid-Spathian^13^—almost all Early Triassic marine reptiles are from the Subcolumbites Zone, which is fourth from the bottom of the five Spathian ammonite zone, while one is known from the underlying Procolumbites Zone (see Supplementary Note). This indicates a rapid diversification of prey preference in less than one million years.

The presence/absence of feeding categories in Supplementary Table S1 reveals three different patterns of stratigraphic distribution. The first pattern is the consistent presence of the same feeding mode throughout the Triassic, which is represented by two categories: pelagic ram feeders with pointed teeth (mostly ichthyopterygians), and demersal predators with round teeth (placodont sauropterygians and thalattosaurs). The second pattern is characterized by first appearance in the Early Triassic and disappearance at various times within the Early or Middle Triassic. Four categories belong to this pattern: pelagic ram feeders with round teeth (omphalosaurs), demersal ram feeders with pointed teeth (basal eosauropterygians), demersal ram feeders without teeth (hupehsuchians), and a demersal suction feeder without teeth (*Cartorhynchus*). The third pattern is a combination of appearance at various points in time during the Middle and Late Triassic and a short stratigraphic span of no more than a substage. This pattern includes a pelagic ram feeder without teeth (*Guanlingsaurus*), demersal ram feeder with filters (*Atopodentatus*), and demersal ram feeder without teeth (*Endennasaurus*).

Overall, the diversification of feeding strategies among marine tetrapods in the Triassic can be seen as an early burst in the Early Triassic followed by gradual decrease (Fig. 4A). Thus, feeding strategy diversity in the Early Triassic is exceptionally high for marine reptiles in comparison with those shown in their later Triassic history. Those taxa with the first two patterns of stratigraphic distribution listed above represent the early burst. Opportunities for a limited number of new feeding strategies also emerged in the Middle and Late Triassic. These new strategies add noise to the major pattern identified but do not alter the larger picture, probably because none of these new strategies lasted for more than a geologic stage.

The incompleteness of the fossil record may provide a caveat to the foregoing discussion. The fossil record is inherently biased by such factors as the availability of fossil-bearing rock exposures, relative concentrations of fossils in those rocks, and collection intensity. Therefore, the apparent stratigraphic range of each feeding type may be shorter than it really was. Many methods have been proposed to account for the biases with regards to the stratigraphic range of a taxon^44^. However, most of these methods are designed to apply to a single stratigraphic section, rather than to the global fossil record. Moreover, data available for fossil marine reptiles are not sufficiently detailed to allow meaningful error range estimates. Furthermore, use of normal distributions would likely lead to overestimation of error range in the present case, where 94.4% of about 150 species of Triassic marine reptiles with unambiguous stratigraphic provenance are known only from one geologic substage. Nevertheless, to account for the possible influence of the incompleteness of the fossil record, we examined a hypothetical inflation of stratigraphic ranges where each feeding type appeared one stage earlier and lasted one stage longer than the actual fossil record (Fig. 4B). We made an exception to this stratigraphic range inflation for the very first appearance in the Spathian, which is very unlikely to extend back to the Smithian^13^. This procedure is most likely an overcompensation for the missing records but at least serves as an extreme case to bracket our interpretation. However, as seen in Fig. 4B, this extreme treatment did not alter any of the major patterns that we discussed earlier. Note that it is not feasible to correct the total count of feeding types per substage directly, as in taxonomic diversity curve correction, without considering the stratigraphic range of each type first. Unlike taxonomic counts that may be much larger than what is preserved in the fossil record, the total number of feeding types is probably not much greater than nine because about half of the 24 possible feeding types are unlikely to be occupied by marine tetrapods. If, for example, one triples the feeding-type diversity in the Bithynian considering the scarcity of appropriate rock exposures, the total count would exceed nine.

### Implications for trophic structure evolution

The feeding style diversity of Triassic marine reptiles provides a unique tool to examine the biotic recovery of their prey. These predators collected live prey, including those that are not usually preserved in the fossil record. There is a clear tendency for the fossil record to preserve more hard- than soft-bodied animals, while these predators presumably fed on both. Also, marine reptiles may have hunted in habitats or areas that are not preserved in accessible geologic strata, thus providing insight into prey that is geographically hidden from the fossil record.

As pointed out earlier, it is generally thought that marine trophic structure recovered from the end-Permian extinction slowly and gradually toward the mid-Middle Triassic^2^. The pattern we describe above for the evolution of feeding guilds among marine reptiles does not support this conclusion. The variety of prey consumed by these predators was most likely greatest in the Early Triassic, given the high diversity of their feeding styles. This in turn suggests that the greatest variety of prey was already present in the Early Triassic, contrary to the model of slow, gradual recovery.

One possible reason for the difference in the suggested recovery rates may be that the recovery of prey that are unpreserved as fossils preceded that of their preserved counterparts, and that early marine reptiles were feeding on the former. In addition to soft-bodied animals, some arthropods may not be preserved because their exoskeletons are not calcified unlike in bivalves, gastropods, or brachiopods. Supporting such a view is the nature of the feeding strategies unique to the Early Triassic, namely demersal suction feeding in *Cartorhynchus* and lunge feeding in Hupehsuchia. Both taxa are edentulous, small, and heavily built for feeding near the seafloor. As reported elsewhere, no fish are known from the Nanzhang-Yuan'an Fauna, where hupehsuchians are found. Some Spathian horizons in Nanzhang-Yuan'an are heavily bioturbated, indicating the presence of abundant soft-bodied infaunal and epifaunal invertebrates, while other horizons have conodonts. Given the high diversity of hupehsuchians despite the lack of hard-bodied prey organisms, it is most likely that they fed at least partly on soft-bodied prey. Extant lunge feeders are known to feed on crustaceans, although the proportion of crustaceans in the diet is limited in pelicans. The Chaohu fauna that contains *Cartorhynchus* has some records of co-occurring invertebrates, including hooklets of soft-bodied coleoids and thylacocephalans. However, *Cartorhynchus* is small and not equipped to feed on any of the invertebrates that co-occur with it. It therefore fed on organisms that are not preserved as fossils, including soft-bodied animals and possibly some crustaceans.

### Nutrient cycling and ‘recovery'

The appearance of marine tetrapods in the Early Triassic added a new pathway for nutrients to circulate in coastal waters. The modern ‘Whale Pump' refers to the cycling of nutrients, especially nitrogen, to the euphotic zone from the layers below, facilitated by large marine mammals that feed below the euphotic zone and defecate near the sea surface^6^. Small marine mammals, such as harbour seals and porpoises, also contribute to this activity^6^. The diving abilities of these small marine mammals are limited but coastal euphotic zones are shallow^45^. Even if they do not forage below the euphotic zone, it is known that marine tetrapods still contribute to the re-distribution of nutrients, especially iron, in the modern Southern Ocean^7^ by feeding at various depths and defecating near the surface, where the primary productivity is highest^5^. Bioturbation on the seafloor together with the upward transport of nutrients by the tetrapods are processes that potentially enrich the plankton and make the water column more productive. It is possible that early marine tetrapods also contributed to the cycling of these nutrients at least to some extent.

Predation on demersal-feeding marine tetrapods by other marine tetrapods would also enhance such nutrient cycling, even if predation occurred near the sea surface. There was a high diversity of marine reptiles in the Early Triassic, and some of them were small enough to be consumed by others. Hupehsuchians, which coexisted with larger sauropterygians, evolved a high level of body protection, including a strange bony body tube^15^, and one specimen shows a damaged paddle that was most likely bitten off by a predator^16^. A large humerus from the Early Triassic of Idaho likely belongs to a large ichthyopterygian^3^, which was probably sufficiently large to consume smaller marine reptiles. These pieces of evidence suggest that predation of marine tetrapods already began in the Early Triassic, and likely enhanced the nutrient cycling efficiency.

Given that nutrient pumping by marine tetrapods did not exist in the Late Permian, the marine trophic structure after the appearance of these animals in the Early Triassic was not equivalent to the pre-extinction trophic structure. The effect of nutrient cycling was probably limited at the beginning, especially globally. However, at least some locations, such as Chaohu and Nanzhang-Yuan'an, likely had a large number of marine tetrapods given the abundant fossil record from these locations. The latest Early Triassic seems to mark the time when the new trophic structure became apparent.

## Methods

### Definition of lunge feeding

Lunge feeding refers to the feeding style of rorqual whales (Balaenopteridae) that use a distensible gular pouch and mandibular widening^35^,^46^,^47^. The term has also been used for a completely different feeding style that involved lunging^48^ but it has not been used in this second context for more than two decades. During the lunge feeding of rorqual whales, food items are first captured with a large amount of water in a gular pouch, containing ventral groove blubber that stretches extensively^49^. This expansion of the gular pouch is associated with widening of the mandible through rotation of each ramus. The water is later expunged through filters with help from the tongue, leaving the prey in the mouth cavity^20^. Lunge feeding is a specialized strategy involving many modifications of the relevant body parts^47^,^50^. It is a putative key innovation that led to the success of rorqual whales^20^.

A similar feeding mechanism using a gular pouch and mandibular widening is known in pelicans^51^, whose feeding style is usually called plunge feeding because they plunge into the water from the air. The mandibular rami of pelicans bow strongly as the gular pouch expands to capture prey together with the surrounding water^22^, which is gravitationally drained by raising the beak above the water surface^51^. In contrast, the strongly curved mandibular rami of rorquals do not bow but are rotated in a complex manner to achieve a similar effect of expanding the gular space^19^, and the water is actively expelled. Despite the differences, the rostral mechanics of pelicans and rorquals are the closest analogs with each other, and resulted in similar mechanical constraints on mandibular shape^19^.

It has been suggested that the pelican eel (*Eurypharynx pelecanoides*) also feeds in a manner similar to that of pelicans. However, to our knowledge, direct observation of feeding by this species has not been reported in the scientific literature. The mandible widens greatly from the natural posture during feeding, and there are distensible gular and buccal pouches.

There is no simple name to refer to the feeding style that is common among pelicans, rorquals, and the pelican eel, which all involve mandibular widening and gular pouch expansion to capture prey with the surrounding water. Rather than inventing a new terminology, we refer to it as lunge feeding in the present paper. Lunge feeding is used for suspension feeding in some whales and the pelican eel, and macrophagy in pelicans and whales. It has not been common throughout the history of life, with virtually no fossil record of this feeding behaviour outside of these groups. Cretaceous stomatosuchid crocodyliforms^52^ and the pterosaur *Ikrandraco*^53^ were probably not lunge feeders in the sense discussed in the present paper, given their solid jaw symphyses that prevent mandibular widening (see also Fig. 2K–M). Thus, apart from *Hupehsuchus*, lunge feeding appears to be a unique feature of the modern ecosystem, appearing at most as early as the late Oligocene (about 30 million years ago) in the earliest pelicans^54^, and about 20 million years ago in Balaenopteridae^55^,^56^.

### Mandible shape

We compared the lateral profiles of 30 tetrapod mandibles to examine their similarity to *Hupehsuchus* (see Fig. 2 for the list of species). The exact flexural rigidity distribution within a mandibular ramus cannot be easily estimated in fossils. We instead approximated the distribution of flexural rigidity relative to the mandibular length, based on the lateral profile of the jaw. We assumed that the transverse sections are roughly oval rings with a constant proportion of the center space, and that the elastic modulus does not change drastically along the jaw. Then, flexural rigidity at a given point along a mandibular ramus is proportional to the biquadratic of the ramus depth at each point (Fig. 3A). Given the approximation that we stated above, we compared the resulting relative rigidity distribution for a pelican with the published curve. The result (Fig. 3B) suggests that the approximation can at least recover major patterns in the flexural rigidity distribution, although it may overestimate the rigidity near the deepest parts, where bony walls are thinner than the rest, while underestimating the rigidity in shallow parts, where the bones tend to leave less open space.

The lateral profile of each mandible was outlined in CorelDraw and converted to a bitmap that spans 1500 pixels. The profiles were taken from the literature listed in the caption to Figure 2. The outline was digitized as Cartesian coordinates in ImageJ^57^, resulting in mandibular outline coordinates in each of the 1500 slices. The mean depth of mandible for each taxon, given in Fig. 2, was calculated as the average of 1500 depths resulting from these coordinates. The resolution along the longitudinal axis was then reduced to 1/20 to enable computation, giving rise to coordinates of the top and bottom margins of the mandible at every 1/75th point along the mandible (150 coordinates in total). These coordinates were used to derive mandibular depth at every 1/74th point of the mandible.

The same coordinates were used as pseudo-landmarks in a geometric morphometric analysis. These pseudo-landmarks are not homologous across specimens. However, from a perspective of flexural rigidity, they represent approximately equivalent points along the mandible. It is difficult to find homologous landmarks, especially of type 1, across the taxa being examined, but given that our interest is in flexural rigidity and not taxonomy, it seems plausible to use these coordinates in the present case. We applied Procrustes transformation to the coordinates and then ran a Principal Component Analysis (PCA) in the geomorph^58^ package of R. PCA axes were interpreted based on thin plate spline warping (Fig. 3C). To summarize the signals in different PC axes, including the ones not depicted in Figure 3C, a hierarchical cluster analysis was applied on all PC scores to illuminate similarities in mandibular shape, based on Euclid distances and Ward method. (Fig. 3D). Only those taxa with the mean mandibular depth equal to or lower than 3.0% of the mandibular length were included in the geometric morphometric analysis—inclusion of robust jaws resulted in confusing signals arising from jaw-depth variation in the data set.

### Mandible size

We used data compiled mainly from one source^35^, added one data point from another^59^, and logarithmically transformed the values for allometric analysis. Species means, rather than raw data, were used. Means were calculated as geometric means, which are the same as averaging log-transformed values, to account for the bias on arithmetic means from scaling effects within each species. Phylogenetic generalized least square (PGLS), as well as standardized major axis based on phylogenetically independent contrasts (SMA-PIC) were used for linear regressions. We used the phylogeny and molecular divergence time estimate by^56^ for phylogenetic bias removal, assuming Brownian motion. All calculations were performed in R^60^. See Supplementary Note for the list of R packages used.

### Feeding guilds of fossil marine tetrapods

We used the following three criteria to categorize fossil marine tetrapods into 24 different feeding guilds. Habitat (pelagic or demersal)—clades with pachyostosis, heavy gastral basket, or a device to interact with substrates (e.g., procumbent rostra of thalattosaurs) were considered to be demersal, and the rest pelagic. Prey capture (ram with biting, lunge, or suction)—clades with a suite of hyoid and rostral features that are mechanically important for suction feeding^37^ were considered suction feeders, those with features described in this paper as lunge feeders, and the rest ram feeders that capture prey between the jaws. Lunge feeding is a kind of ram feeding but enables suspension feeding, and therefore was placed in a separate category. Tooth shape (point, round, filter, or edentulous)—we referred to the shape of a typical tooth (usually the largest one) among the dentition following a previous author^42^.

## Author Contributions

R.M. conceived the study, observed the specimen, ran analyses, drew figures, and wrote the manuscript. X.C. collected the fossil, observed it, and revised the manuscript. D.J. conceived the study, observed the specimen, and revised the manuscript. L.C. collected the fossil and observed the specimen. A.T. conceived the study and revised the manuscript. O.R. conceived the study and revised the manuscript.

## Supplementary Material

Supplementary Information Supplementary Information
