# Supplementary material for: Lunge feeding in early marine reptiles and fast evolution of marine tetrapod feeding guilds
Source: Sci Rep. 2015 Mar 10;5:8900. doi: 10.1038/srep08900 (PMC4354009; doi:10.1038/srep08900)
Supplement: Supplementary Information [file srep08900-s1.pdf]

Supplementary Information for:

Lunge feeding in early marine reptiles and fast evolution of marine tetrapod feeding guilds

RYOSUKE MOTANI[*,1], XIAO-HONG CHEN[*,2], DA-YONG JIANG[3], LONG CHENG[2], ANDREA TINTORI[4], and OLIVIER RIEPPEL[5]

[1]Department of Earth and Planetary Sciences, University of California, Davis, One Shields Avenue, Davis, California 95616, U.S.A.;

[2]Wuhan Centre of China Geological Survey, Wuhan, Hubei 430023, P. R. China.

[3]Laboratory of Orogenic Belt and Crustal Evolution, Ministry of Education; Department of Geology and Geological Museum, Peking University, Yiheyuan Street. 5, Beijing 100871, P.R. China;

[4]Dipartimento di Scienze della Terra, Università degli Studi di Milano, Via Mangiagalli 34-20133 Milano, Italy

[5]Center of Integrative Research, The Field Museum, Chicago, IL 60605-2496, U. S. A;

[*]Corresponding authors: RYOSUKE MOTANI, rmotani@ucdavis.edu; XIAO-HONG CHEN, yccxiaohong@163.com.

Supplementary Note

A. Permian marine reptile

Mesosaurs are the only group of marine reptiles before the Triassic but their limited geographical and temporal spans prevented them to affect the open marine trophic structure before the end-Permian extinction. These reptiles lived in restricted seas[1], only for a short time span within the Artinskian (Early Permian)[2].

1. Rossmann T. Studies on mesosaurs (Amniota inc. sed., Mesosauridae): 3. New aspects on the anatomy, preservation and palaeoecology, based on the specimens from the Palaeontological Institute of the University of Zurich. *Neues Jahrb Geol P-A* **224**, 197-221 (2002).
2. Holz M, Franca AB, Souza PA, Iannuzzi R, Rohn R. A stratigraphic chart of the Late Carboniferous/Permian succession of the eastern border of the Parana Basin, Brazil, South America. *J S Am Earth Sci* **29**, 381-399 (2010).

B. Triassic marine reptiles before mid-Spathian.

There is no Triassic marine reptiles that is known to be definitively older than the mid-Spathian. Almost all Early Triassic marine reptile records are from the Subcolumbites Zone, which is the fourth from the bottom of the five ammonite zones of the Spathian[1], when known. This is true for Anhui[2], South Kitakami[3], and Spitsbergen[4]. Specimens from British Columbia[5, 6, 7, 8] and Idaho[9, 10] are from screes, while *Thaisaurus* from Thailand has unknown age within the Triassic[11]. *Corosaurus* from Wyoming has a controversial stratigraphic position that could be anywhere in the Anisian (Middle Triassic) and Olenekian (Lower Triassic) because of disconformity and lack of index fossils.

Some of thalattosaurs[6] and ichthyosaurs[8] from British Columbia were once considered to be from the Smithian but this idea was later overturned by the original

authors[7], who considered them to be younger. The only record definitively older than the Subcolumbites Zone is a *Chaohusaurus* from the Procolumbites Zone (mid-Spathian)[2].

1. Gradstein FM, Ogg JG, Schmitz MD, Ogg GM. *The Geologic Time Scale 2012*. Elsevier (2012).
2. Motani R, Jiang D, Tintori A, Rieppel O, Chen GB. Terrestrial origin of viviparity indicated by the oldest embryonic fossil of Mesozoic marine reptiles. *PLoS One* **9**, e8B640 (2014).
3. Ehiro M. Spathian ammonoids *Metadagnoceras* and *Keyserlingites* from the Osawa Formation in the Southern Kitakami Massif, Northeast Japan. *Trans Proc Palaeontol Soc Japan New Ser* **171**, 229-236 (1993).
4. Harland WB. The geology of svalbard. *Memoir of the Geological Society of London* **17**, 1-521 (1997).
5. Brinkman DB, Zhao XJ, Nicholls EL. A Primitive Ichthyosaur from the Lower Triassic of British-Columbia, Canada. *Palaeontology* **35**, 465-474 (1992).
6. Nicholls EL, Brinkman D. New thalattosaurs (Reptilia: Diapsida) from the Triassic Sulphur Mountain Formation of Wapiti Lake, British Columbia. *Journal of Paleontology* **67**, 263-278 (1993).
7. Nicholls EL, Brinkman DB. A new ichthyosaur from the Triassic of Sulphur Mountain Formation of British Columbia. In: *Vertebrate Fossils and the Evolution of Scientific Concepts* (ed^(eds Sarjeant WAS). Gordon and Breach (1995).
8. Callaway JM, Brinkman DB. Ichthyosaurs (Reptilia, Ichthyosauria) from the Lower and Middle Triassic Sulfur Mountain Formation, Wapiti Lake Area, British-Columbia, Canada. *Can J Earth Sci* **26**, 1491-1500 (1989).
9. Massare JA, Callaway JM. Cymbospondylus (Ichthyosauria, Shastasauridae) from the Lower Triassic Thaynes Formation of Southeastern Idaho. *Journal of Vertebrate Paleontology* **14**, 139-141 (1994).
10. Scheyer TM, Romano C, Jenks J, Bucher H. Early Triassic marine biotic recovery: the predators' perspective. *PLoS One* **9**, e88987 (2014).
11. McGowan C, Motani R. *Ichthyopterygia*. Verlag Dr. Friedrich Pfeil (2003).

C. Effect of metabolic rate on the relative skull size of lunge feeders.

The difference in metabolic rates between mammals and reptiles may lead to different size of lunge feeding apparatuses, especially the mandible. We accounted for this bias by using an approximate model below. The weak positive allometry observed in mandibular length is expected based on a simple dimensional argument. It is generally

known that the basal metabolic rates (Mb) are approximately proportional to the body mass (W) raised to 0.75, although controversies exist. Assuming that the body mass is roughly proportional to the cube of body length (Lb) in similarly shaped animals:

$$Mb = c_1W^{0.75} = c_1c_2Lb^{2.25} \quad (1)$$

where c_1 and c_2 are constants. The amount of food that passes through the gape per time is proportional to the cross sectional area of the gape, which is roughly proportional to the square of mandibular length (Lm), assuming a limited variation in jaw design, including gape angle. Then, if food consumption rate is roughly proportional to the basal metabolic rate and foraging duration does not differ drastically among individuals:

$$Mb = c_3Lm^2 \quad (2)$$

where c_3 is a constant. Then, from (1) and (2):

$$Lm = (c_1c_2)^{0.5}c_3^{-1}Lb^{1.125} \quad (3).$$

This model involves many approximations and therefore is admittedly simplistic. Yet, the expected slope of 1.125 is similar to 1.07 reported in this paper, and 1.11 previously[1]. In equation (3), the difference in metabolic rates is represented by a constant c_1. Then, the difference in mandibular length is expected to be roughly proportional to the square root of the difference in metabolic rates.

The model described above suggests that reptiles should have smaller mandibles for lunge feeding than mammals because of their lower metabolic rates that would mandate less food consumption, everything else being the same. At 37°C, average mammals would use about 7 times more oxygen than the average reptiles of the same size, based on the data compiled by[2] and the equations of[3]. Then, from equation (3) in Methods, average reptiles

would have about 38% the mandibular length of average mammals for lunge feeding, where mandibular size is expected to determine the amount of water captured per time for a given relative speed. If so, an average lunge-feeding reptile at the size of *Hupehsuchus* would have a mandibular length of about 6.19 cm. The observed length of 13.34 cm is much longer than this expected value. A combination of many factors can explain the difference between the two values, such as lower prey density, less foraging time, and higher-than-average metabolic rate for *Hupehsuchus*. None of these factors, however, can be estimated reasonably. In conclusion, the skull of *Hupehsuchus* is not smaller than what is expected for a lunge-feeding reptile of its size.

1 Pyenson, N. D., Goldbogen, J. A. & Shadwick, R. E. Mandible allometry in extant and fossil Balaenopteridae (Cetacea: Mammalia): the largest vertebrate skeletal element and its role in rorqual lunge feeding. *Biological Journal of the Linnean Society* **108**, 586-599, doi:DOI 10.1111/j.1095-8312.2012.02032.x (2013).

2 Makarieva, A. M. *et al.* Mean mass-specific metabolic rates are strikingly similar across life's major domains: Evidence for life's metabolic optimum. *Proceedings of the National Academy of Sciences of the United States of America* **105**, 16994-16999, doi:DOI 10.1073/pnas.0802148105 (2008).

3 Gillooly, J. F., Brown, J. H., West, G. B., Savage, V. M. & Charnov, E. L. Effects of size and temperature on metabolic rate. *Science* **293**, 2248-2251, doi:DOI 10.1126/science.1061967 (2001).

D. R packages used.

1 Garland, T., Harvey, P. H. & Ives, A. R. Procedures for the Analysis of Comparative Data Using Phylogenetically Independent Contrasts. *Systematic Biol* **41**, 18-32, doi:10.2307/2992503 (1992).

2 R+Core+Team. *R: A language and environment for statistical computing.* . (R Foundation for Statistical Computing, Vienna, Austria. URL http://www.R-project.org/, 2013).

3 Paradis, E., Claude, J. & Strimmer, K. APE: Analyses of Phylogenetics and Evolution in R language. *Bioinformatics* **20**, 289-290, doi:10.1093/bioinformatics/btg412 (2004).

4 Warton, D. I., Duursma, R. A., Falster, D. S. & Taskinen, S. smatr 3-an R package for estimation and inference about allometric lines. *Methods Ecol Evol* **3**, 257-259, doi:10.1111/j.2041-210X.2011.00153.x (2012).

E. References used for Figure 2.

1 McGowan, C. & Motani, R. *Ichthyopterygia*. Vol. 8 (Verlag Dr. Friedrich Pfeil, 2003).

2 Motani, R. *et al.* A basal ichthyosauriform with a short snout from the Lower Triassic of China. *Nature* (in press).

3 Nielsen, J. G., Bertelsen, E. & Jespersen, A. The Biology of *Eurypharynx pelecanoides* (Pisces, Eurypharyngidae). *Acta Zool-Stockholm* **70**, 187-197 (1989).

4 Meyers, R. A. & Myers, R. P. Mandibular bowing and mineralization in Brown Pelicans. *Condor* **107**, 445-449, doi:10.1650/7743 (2005).

5 Ferry-Graham, L. A., Wainwright, P. C., Hulsey, C. D. & Bellwood, D. R. Evolution and mechanics of long jaws in butterflyfishes (family Chaetodontidae). *J Morphol* **248**, 120-143, doi:10.1002/Jmor.1024 (2001).

6 Kammerer, C. F., Grande, L. & Westneat, M. W. Comparative and developmental functional morphology of the jaws of living and fossil gars (Actinopterygii : Lepisosteidae). *J Morphol* **267**, 1017-1031, doi:10.1002/Jmor.10293 (2006).

7 Montgomery, J. C. & Saunders, A. J. Functional-Morphology of the Piper Hyporhamphus-Ihi with Reference to the Role of the Lateral Line in Feeding. *Proc R Soc Ser B-Bio* **224**, 197-208, doi:10.1098/rspb.1985.0029 (1985).

8 Zusi, R. L. Introduction to the skeleton of hummingbirds (Aves: Apodiformes, Trochilidae) in functional and phylogenetic contexts. *Ornithological Monographs* **77**, 1-94 (2013).

9 Shufeldt, R. W. Osteology of *Numenius longirostris*, with notes upon the skeletons of other American Limicolae. *J Anat Physiol* **19**, 1-93 (1884).

10 Ferreira, C. D. & Donatelli, R. J. Skull osteology of Platalea ajaja (Linnaeus) (Aves, Ciconiiformes), compared with others species of Threskiornithidae. *Rev Bras Zool* **22**, 529-551, doi:10.1590/S0101-81752005000300003 (2005).

11 Sereno, P. C. & Larsson, H. C. E. Cretaceous Crocodyliforms from the Sahara. *Zookeys*, 1-143, doi:10.3897/zookeys.28.325 (2009).

12 Wang, X., Rodrigues, T., Jiang, S., Cheng, X. & Kellner, A. W. A. An Early Cretaceous pterosaur with an unusual mandibular crest from China and a potential novel feeding strategy. *Scientific Reports* **4**, 6329, doi:10.1038/srep06329 (2014).

13 Motani, R., Jiang, D., Tintori, A., Rieppel, O. & Chen, G. B. Terrestrial origin of viviparity indicated by the oldest embryonic fossil of Mesozoic marine reptiles. *PLoS One* **9**, e8B640, doi:10.1371/journal.pone.0088640 (2014).
14 Shufeldt, R. W. Comparative osteology of certain rails and cranes, and the systematic positions of the supersuborders Gruiformes and Ralliformes. *Anatomical Record* **9**, 731-750, doi:10.1002/ar.1090091002 (1915).
15 Johnson, R. The Cranial and Cervical Osteology of the European Oystercatcher Haematopus-Ostralegus L. *J Morphol* **182**, 227-244, doi:DOI 10.1002/jmor.1051820209 (1984).
16 Raikow, R. J. Osteology and Taxonomic Position of White-Backed Duck, Thalassornis-Leuconotus. *Wilson Bull* **83**, 270-& (1971).
17 Shufeldt, R. W. *Osteology of Birds*. (University of the State of New York, 1909).

Supplementary Table S1. Feeding type diversity of marine reptiles in the Triassic. Brackets suggest that a feeding style was necessarily present because the following clades persisted through the interval despite the lack of direct record: Pelagic-ram-pointed, Ichthyoptrygia; Pelagic-ram-rounded, Omphalosauridae; Demersal-ram-pointed, Eosauropterygia; and Demesal-ram-rounded, Placodontia.

Habitat	Pelagic			Demersal						
Capture	ram with biting							suction	lunge	
Teeth	p	r	n	p	r	n	f'	n	n/f	Total
Rhaetian	X				X					2
Sevatian	(X)				(X)					2
Alaunian	X				X	X				3
Lancian	X				(X)					2
Tuvalian	X				X					2
Julian	X		X	X	X					4
Longobardian	X			X	X					3
Fassanian	X	X		X	X					4
Illyrian	X	X		X	X					4
Pelsonian	X	X		X	X		X			5
Bithynian	X	(X)		(X)	X					4
Aegean	(X)	(X)		X	X					4
Spathian	X	X		X	X			X	X	6

Supplementary Table S2. Data used to make Supplementary Table S1. Stratigraphic range was derived from a Kelley et al. (2014)[1] for most species; references are given for species that we added. In a trial to maximize the number of feeding types per substage, age assignments that were found to be questionable by Kelley et al. (2014) were all considered valid. For the same reason, taxa that span two substages were counted for both substages.

Binomial	CLADE	Habitat	Approach	Teeth	FirstSubstage	LastSubstage
Ichthyosaurus communis	Ichthyopterygia	pelagic	ram	point	Rhaetian	Early Sinemurian
Macroplacus raeticus	Sauropterygia	demersal	ram	round	Rhaetian	Rhaetian
Psephoderma anglicum	Sauropterygia	demersal	ram	round	Rhaetian	Rhaetian
Callawayia neoscapularis	Ichthyopterygia	pelagic	ram	point	Lacian	Lacian
Hudsonelpidia brevirostris	Ichthyopterygia	pelagic	ram	point	Lacian	Lacian
Sikannisuchus huskyi	Archosauria	pelagic	ram	point	Lacian	Lacian
Himalayasaurus tibetensis	Ichthyopterygia	pelagic	ram	point	Lacian/Alaunian	Lacian/Alaunian
Endennasaurus acutirostris	Thalattosauriformes	demersal	ram	none	Alaunian	Alaunian
Macgowania janiceps	Ichthyopterygia	pelagic	ram	point	Alaunian	Alaunian
Psephoderma alpinum	Sauropterygia	demersal	ram	round	Alaunian	Rhaetian
Shonisaurus sikanniensis	Ichthyopterygia	pelagic	ram	point	Alaunian	Alaunian
Californosaurus perrini	Ichthyopterygia	pelagic	ram	–	Tuvalian	Tuvalian
Nectosaurus halinus	Thalattosauriformes	demersal	ram	round	Tuvalian	Tuvalian
Shastasaurus pacificus	Ichthyopterygia	pelagic	ram	point	Tuvalian	Tuvalian
Shonisaurus popularis	Ichthyopterygia	pelagic	ram	point	Tuvalian	Tuvalian
Thalattosaurus alexandrae	Thalattosauriformes	demersal	ram	round	Tuvalian	Tuvalian
Toretocnemus californicus	Ichthyopterygia	pelagic	ram	point	Tuvalian	Tuvalian
Toretocnemus zitteli	Ichthyopterygia	pelagic	ram	point	Tuvalian	Tuvalian
Placochelys placodonta	Sauropterygia	demersal	ram	round	Julian/Tuvalian	Julian/Tuvalian
Anshunsaurus huangguoshuensis	Thalattosauriformes	pelagic	ram	point	Julian	Julian
Bobosaurus forojuliensis	Sauropterygia	pelagic	ram	–	Julian	Julian
Concavispina biseridens[2]	Thalattosauriformes	demersal	ram	round	Julian	Julian

Guanlingsaurus liangae	Ichthyopterygia	pelagic	ram	none	Julian	Julian
Guizhouichthyosaurus tangae	Ichthyopterygia	pelagic	ram	point	Julian	Julian
Henodus chelyops	Sauropterygia	demersal	ram	round	Julian	Julian
Miodentosaurus brevis	Thalattosauriformes	pelagic	ram	point	Julian	Julian
Protenodontosaurus italicus	Sauropterygia	demersal	ram	round	Julian	Julian
Psephochelys polyosteoderma	Sauropterygia	demersal	ram	round	Julian	Julian
Qianichthyosaurus zhoui	Ichthyopterygia	pelagic	ram	point	Julian	Julian
Sinocyamodus xinpuensis	Sauropterygia	demersal	ram	round	Julian	Julian
Xinpusaurus bamaolinensis	Thalattosauriformes	demersal	ram	round	Julian	Julian
Xinpusaurus suni	Thalattosauriformes	demersal	ram	round	Julian	Julian
Anshunsaurus huangnihensis[3]	Thalattosauriformes	pelagic	ram	point	Longobardian	Longobardian
Anshunsaurus wushaensis	Thalattosauriformes	pelagic	ram	point	Longobardian	Longobardian
Fuyuansaurus acutirostris[4]	Protorosauria	pelagic	ram	point	Longobardian	Longobardian
Glyphoderma kangi	Sauropterygia	demersal	ram	round	Longobardian	Longobardian
Keichousaurus hui	Sauropterygia	demersal	ram	point	Longobardian	Longobardian
Lariosaurus balsami	Sauropterygia	demersal	ram	point	Longobardian	Longobardian
Lariosaurus valceresii	Sauropterygia	demersal	ram	point	Longobardian	Longobardian
Lariosaurus xingyiensis	Sauropterygia	demersal	ram	point	Longobardian	Longobardian
Macrocnemus fuyuanensis	Protorosauria	pelagic	ram	point	Longobardian	Longobardian
Neusticosaurus staubi	Sauropterygia	demersal	ram	point	Longobardian	Longobardian
Neusticosaurus toeplitschi	Sauropterygia	demersal	ram	point	Longobardian	Longobardian
Nothosaurus cymatosauroides	Sauropterygia	demersal	ram	point	Longobardian	Longobardian
Nothosaurus edingerae	Sauropterygia	demersal	ram	point	Longobardian/Julian	Longobardian/Julian
Nothosaurus youngi	Sauropterygia	demersal	ram	point	Longobardian	Longobardian
Psephosaurus suevicus	Sauropterygia	demersal	ram	round	Longobardian	Longobardian

Qianichthyosaurus xingyiensis[5]	Ichthyopterygia	pelagic	ram	point	Longobardian	Longobardian
Qianxisaurus chajiangensis[6]	Sauropterygia	demersal	ram	point	Longobardian	Longobardian
Yunguisaurus liae	Sauropterygia	pelagic	ram	point	Longobardian	Longobardian
Lariosaurus curionii	Sauropterygia	demersal	ram	point	Fassanian/Longobardian	Fassanian/Longobardian
Askeptosaurus italicus	Thalattosauriformes	pelagic	ram	point	Fassanian	Fassanian
Blezingeria ichthyospondylus	Thalattosauriformes		ram	–	Fassanian	Fassanian
Ceresiosaurus calcagnii	Sauropterygia	demersal	ram	point	Longobardian	Fassanian
Ceresiosaurus lanzi	Sauropterygia	demersal	ram	point	Longobardian	Fassanian
Cyamodus hildegardensis	Sauropterygia	demersal	ram	round	Fassanian	Fassanian
Cymbospondylus piscosus	Ichthyopterygia	pelagic	ram	point	Fassanian	Fassanian
Lariosaurus buzzii	Sauropterygia	demersal	ram	point	Fassanian	Fassanian
Neusticosaurus edwardsi	Sauropterygia	demersal	ram	point	Fassanian	Fassanian
Neusticosaurus peyeri	Sauropterygia	demersal	ram	point	Fassanian	Fassanian
Neusticosaurus pusillus	Sauropterygia	demersal	ram	point	Fassanian	Fassanian
Nothosaurus jagisteus	Sauropterygia	demersal	ram	point	Fassanian	Fassanian
Omphalosaurus wolfi	Omphalosauria	pelagic	ram	round	Fassanian	Fassanian
Paraplacodus broilii	Sauropterygia	demersal	ram	round	Fassanian	Fassanian
Serpianosaurus mirigiolensis	Sauropterygia	demersal	ram	point	Fassanian	Fassanian
Simosaurus gaillardoti	Sauropterygia	demersal	ram	point	Fassanian	Julian
"Psephosaurus" sinaiticus	Sauropterygia	demersal	ram	round	Illyrian/Fassanian	Illyrian/Fassanian
Clarazia schinzi	Thalattosauriformes	demersal	ram	round	Illyrian/Fassanian	Illyrian/Fassanian
Eusaurosphargis dalsassoi	Helveticosauridae	demersal	ram	point	Illyrian/Fassanian	Illyrian/Fassanian
Helveticosaurus zollingeri	Helveticosauridae	demersal	ram	point	Illyrian/Fassanian	Illyrian/Fassanian
Hescheleria ruebeli	Thalattosauriformes	demersal	ram	round	Illyrian/Fassanian	Illyrian/Fassanian
Lariosaurus stensioei	Sauropterygia	demersal	ram	point	Illyrian/Fassanian	Illyrian/Fassanian
Macrocnemus bassanii	Protorosauria	pelagic	ram	point	Illyrian/Fassanian	Illyrian/Fassanian

Nothosaurus haasi	Sauropterygia	demersal	ram	point	Illyrian/Fassanian	Illyrian/Fassanian
Nothosaurus tchernovi	Sauropterygia	demersal	ram	point	Illyrian/Fassanian	Illyrian/Fassanian
Tanystropheus langobardicus	Protorosauria	pelagic	ram	point	Illyrian/Fassanian	Illyrian/Fassanian
Anarosaurus heterodontus	Sauropterygia	demersal	ram	point	Illyrian	Illyrian
Anarosaurus pumilio	Sauropterygia	demersal	ram	point	Illyrian	Illyrian
Augustasaurus hagdorni	Sauropterygia	pelagic	ram	point	Illyrian	Illyrian
Besanosaurus leptorhynchus	Ichthyopterygia	pelagic	ram	point	Illyrian	Illyrian
Cyamodus kuhnschnyderi	Sauropterygia	demersal	ram	round	Illyrian	Fassanian
Cyamodus muensteri	Sauropterygia	demersal	ram	round	Illyrian	Illyrian
Cyamodus rostratus	Sauropterygia	demersal	ram	round	Illyrian	Illyrian
Cymbospondylus buchseri	Ichthyopterygia	pelagic	ram	point	Illyrian	Illyrian
Cymbospondylus nichollsi	Ichthyopterygia	pelagic	ram	point	Illyrian	Illyrian
Dactylosaurus gracilis	Sauropterygia	demersal	ram	point	Illyrian	Illyrian
Mixosaurus cornalianus	Ichthyopterygia	pelagic	ram	round	Illyrian	Illyrian
Mixosaurus kuhnschnyderi	Ichthyopterygia	pelagic	ram	round	Illyrian	Illyrian
Nothosaurus giganteus	Sauropterygia	demersal	ram	point	Illyrian	Julian
Nothosaurus juvenilis	Sauropterygia	demersal	ram	point	Illyrian	Illyrian
Nothosaurus marchicus	Sauropterygia	demersal	ram	point	Illyrian	Illyrian
Nothosaurus mirabilis	Sauropterygia	demersal	ram	point	Illyrian	Fassanian
Omphalosaurus nevadanus	Omphalosauria	pelagic	ram	round	Illyrian	Illyrian
Phalarodon callawayi	Ichthyopterygia	pelagic	ram	round	Illyrian	Illyrian
Phantomasaurus neubigi	Ichthyopterygia	pelagic	ram	point	Illyrian	Illyrian
Pistosaurus longaevus	Sauropterygia	pelagic	ram	–	Illyrian	Illyrian
Atopodentatus unicus	Sauropterygia	demersal	ram	filter	Pelsonian	Pelsonian
Diandongosaurus acutidentatus[7]	Sauropterygia	demersal	ram	point	Pelsonian	Pelsonian
Dianopachysaurus dingi[8]	Sauropterygia	demersal	ram	point	Pelsonian	Pelsonian

Dinocephalosaurus orientalis	Protorosauria	demersal	ram	point	Pelsonian	Pelsonian
Largocephalosaurus plycarpon[9]	Saurosphargidae	demersal	ram	point	Pelsonian	Pelsonian
Largocephalosaurus qianensis[10]	Saurosphargidae	demersal	ram	point	Pelsonian	Pelsonian
Lariosaurus hongguoensis	Sauropterygia	demersal	ram	point	Pelsonian	Pelsonian
Mixosaurus panxianensis	Ichthyopterygia	pelagic	ram	round	Pelsonian	Pelsonian
Mixosaurus xindianensis[11]	Ichthyopterygia	pelagic	ram	round	Pelsonian	Pelsonian
Nothosaurus rostellatus[12]	Sauropterygia	demersal	ram	point	Pelsonian	Pelsonian
Nothosaurus yangjuanensis	Sauropterygia	demersal	ram	point	Pelsonian	Pelsonian
Phalarodon fraasi	Ichthyopterygia	pelagic	ram	round	Pelsonian	Longobardian
Placodus inexpectatus	Sauropterygia	demersal	ram	round	Pelsonian	Pelsonian
Qianosuchus mixtus	Archosauria	pelagic	ram	point	Pelsonian	Pelsonian
Sinosaurosphargis yunguiensis[13]	Saurosphargidae	demersal	ram	point	Pelsonian	Pelsonian
Tanystropheus haasi	Protorosauria	pelagic	ram	point	Pelsonian	Pelsonian
Tholodus schmidi	Ichthyopterygia	demersal	ram	round	Pelsonian	Pelsonian
Wumengosaurus delicatomandibularis	Sauropterygia	pelagic	ram	point	Pelsonian	Pelsonian
Xinminosaurus catactes	Ichthyopterygia	pelagic	ram	round	Pelsonian	Pelsonian
"Psephosaurus" mosis	Sauropterygia	demersal	ram	round	Bithynian	Bithynian
Phalarodon atavus	Ichthyopterygia	pelagic	ram	point	Bithynian	Illyrian
Placodus gigas	Sauropterygia	demersal	ram	round	Bithynian	Fassanian
Chinchenia sungi	Sauropterygia	demersal	ram	point	Aegean_question	Aegean_question
Cymatosaurus fridericianus	Sauropterygia	demersal	ram	point	Aegean	Aegean
Cymatosaurus latifrons	Sauropterygia	demersal	ram	point	Aegean	Aegean
Cymatosaurus minor	Sauropterygia	demersal	ram	point	Aegean	Aegean
Cymatosaurus multidentatus	Sauropterygia	demersal	ram	point	Aegean	Aegean

Germanosaurus latissimus	Sauropterygia	demersal	ram	point	Aegean	Aegean
Germanosaurus schafferi	Sauropterygia	demersal	ram	point	Aegean	Aegean
Nothosaurus winkelhorsti	Sauropterygia	demersal	ram	point	Aegean	Aegean
Nothosaurus winterswjikensis	Sauropterygia	demersal	ram	point	Aegean	Aegean
Sanchiaosaurus dengi	Sauropterygia	demersal	ram	point	Aegean_question	Aegean_question
Thalattosaurus borealis	Thalattosauriformes	demersal	ram	round	Aegean_question	Aegean_question
Agkistrognathus campbelli	Thalattosauriformes	demersal	ram	round	Spathian_question	Spathian_question
Cartorhynchus lenticarpus[14]	Ichthyosauriformes	demersal	suction	none	Spathian	Spathian
Chaohusaurus chaoxianensis	Ichthyopterygia	pelagic	ram	round	Spathian	Spathian
Chaohusaurus geishanensis	Ichthyopterygia	pelagic	ram	round	Spathian	Spathian
Chaohusaurus zhangjiawanensis[15]	Ichthyopterygia	pelagic	ram	round	Spathian	Spathian
Corosaurus alcovensis	Sauropterygia	demersal	ram	point	Spathian	Spathian
Eohupehsuchus brevicollis[16]	Hupehsuchia	demersal	lunge	none	Spathian	Spathian
Grippia longirostris	Ichthyopterygia	pelagic	ram	round	Spathian	Spathian
Gulosaurus helmi[17]	Ichthyopterygia	pelagic	ram	round	Spathian_question	Spathian_question
Hanosaurus hupehensis	Sauropterygia	demersal	ram	point	Spathian	Spathian
Hupehsuchus nanchangensis	Hupehsuchia	demersal	lunge	none	Spathian	Spathian
Isfjordosaurus minor	Ichthyopterygia	pelagic	ram	point	Spathian	Spathian
Keichousaurus yuananensis	Sauropterygia	demersal	ram	point	Spathian	Spathian
Kwangsisaurus orientalis	Sauropterygia	demersal	ram	point	Spathian	Spathian
Majiashanosaurus discocoracoidis[18]	Sauropterygia	demersal	ram	unknown	Spathian	Spathian
Nanchangosaurus suni	Hupehsuchia	demersal	lunge	none	Spathian	Spathian

Omphalosaurus nettarhynchus	Omphalosauria	pelagic	ram	round	Spathian	Spathian
Omphalosaurus nisseri	Omphalosauria	pelagic	ram	round	Spathian	Spathian
Parahupehsuchus longus[19]	Hupehsuchia	demersal	lunge	none	Spathian	Spathian
Paralonectes merriami	Thalattosauriformes	demersal	ram	round	Spathian_question	Spathian_question
Parvinatator wapitiensis	Ichthyopterygia	pelagic	ram	point	Spathian_question	Spathian_question
Thaisaurus chonglakmanii	Ichthyopterygia	pelagic	ram	point	Spathian	Spathian
Utatsusaurus hataii	Ichthyopterygia	pelagic	ram	point	Spathian	Spathian

1. Kelley NP, Motani R, Jiang DY, Rieppel O, Schmitz L. Selective extinction of Triassic marine reptiles during long-term sea-level changes illuminated by seawater strontium isotopes. *Palaeogeogr Palaeocl* **400**, 9-16 (2014).

2. Zhao L-J, Liu J, Li C, He T. A new thalattosaur, *Concavispina iseridens* get. et sp. nov. from Guanling, Guizhou, China. *Vertebrata Palasiatica* **51**, 24-48 (2013).

3. Cheng L, Chen X, Wang C. A new species of Late Triassic *Anshunsaurus* (Reptilia : Thalattosauria) from Guizhou Province. *Acta Geol Sin* **81**, 1345-1351 (2007).

4. Fraser NC, Rieppel O, Chun L. A long-snouted protorosaur from the Middle Triassic of southern China. *Journal of Vertebrate Paleontology* **33**, 1120-1126 (2013).

5. Yang PF, Ji C, Jiang DY, Motani R, Sun ZY. A New Species of Qianichthyosaurus (Reptilia: Ichthyosauria) from Xingyi Fauna (Ladinian, Middle Trassic) of Guizhou, Southwestern China. *Journal of Vertebrate Paleontology* **32**, 197-198 (2012).

6. Cheng YN, Wu XC, Sato T, Shan HY. A new eosauropterygian (Diapsida, Sauropterygia) from the Triassic of China. *Journal of Vertebrate Paleontology* **32**, 1335-1349 (2012).

7. Shang Q-h, Wu X-c, Li C. A new eosauropterygian from Middle Triassic of eastern Yunnan Province, southwestern China. *Vertebrata Palasiatica*, (2011).

8. Liu J*, et al.* A new pachypleurosaur (Reptilia: Sauropterygia) from the lower Middle Triassic of southwestern China and the phylogenetic relationships of Chinese pachypleurosaurs. *Journal of Vertebrate Paleontology* **31**, 292-302 (2011).

9. Cheng L, Chen XH, Zeng XW, Cai YJ. A new eosauropterygian (Diapsida: Sauropterygia) from the Middle Triassic of Luoping, Yunnan Province. *J Earth Sci-China* **23**, 33-40 (2012).

10. Li C, Jiang D, Cheng L, Wu X, Rieppel O. A new species of *Largocephalosaurus* (Diapsida: Saurosphargidae), with implications for the morphological diversity and phylogeny of the group. *Geol Mag* **151**, 100-120 (2014).

11. Chen X-h, Cheng L. A new species of *Mixosaurus* (Reptilia: Ichthyosauria) from the MIddle Triassic of Pu'an, Guizhou, China. *Acta Palaeontologica Sinica* **49**, 251-260 (2010).

12. Shang Q-h. A new species of *Nothosaurus* from the early Middle Triassic of Guizhou, China. *Vertebrata Palasiatica* **44**, 237-249 (2006).

13. Li C, Rieppel O, Wu X, Zhao L, Wang L. A new Triassic marine reptile from southwestern China. *Journal of Vertebrate Paleontology* **31**, 303-312 (2011).

14. Motani R*, et al.* A basal ichthyosauriform with a short snout from the Lower Triassic of China. *Nature* **05 November 2014**, (2014).

15. Chen XH, Sander PM, Cheng L, Wang XF. A New Triassic Primitive Ichthyosaur from Yuanan, South China. *Acta Geol Sin-Engl* **87**, 672-677 (2013).

16. Chen X-h, Motani R, Cheng L, Jiang D-y, Rieppel O. A small short-necked hupehsuchian providing additional evidence of predation on Hupehsuchia. *PLoS One* **9**, e115244 (2014).

17. Cuthbertson RS, Russell AP, Anderson JS. Cranial Morphology and Relationships of a New Grippidian (Ichthyopterygia) from the Vega-Phroso Siltstone Member (Lower Triassic) of British Columbia, Canada. *Journal of Vertebrate Paleontology* **33**, 831-847 (2013).

18. Jiang D*, et al.* Early Triassic eosauropterygian *Majiashanosaurus discocoracoidis*, gen. et sp. nov. (Reptilia, Sauropterygia) from Chaohu, Anhui Province, China. *Journal of Vertebrate Paleontology*, (in press).

19. Chen X, Motani R, Cheng L, Jiang D, Rieppel O. A carapace-like bony 'body tube' in an Early Triassic marine reptile and early onset of marine tetrapod predation. *PLoS One* **9**, e94396 (2014).
